# Acetate transiently inhibits myocardial contraction by increasing mitochondrial calcium uptake

**DOI:** 10.1186/s12899-014-0012-2

**Published:** 2014-12-09

**Authors:** James F Schooley, Aryan M A Namboodiri, Rachel T Cox, Rolf Bünger, Thomas P Flagg

**Affiliations:** Department of Anatomy, Physiology, and Genetics, Uniformed Services University for the Health Sciences, 4301 Jones Bridge Road, Rm. C-2114, Bethesda, 20814 MD USA; Department of Biochemistry and Molecular Biology, Uniformed Services University for the Health Sciences, Bethesda, 20814 MD USA

## Abstract

**Background:**

There is a close relationship between cardiovascular disease and cardiac energy metabolism, and we have previously demonstrated that palmitate inhibits myocyte contraction by increasing K_v_ channel activity and decreasing the action potential duration. Glucose and long chain fatty acids are the major fuel sources supporting cardiac function; however, cardiac myocytes can utilize a variety of substrates for energy generation, and previous studies demonstrate the acetate is rapidly taken up and oxidized by the heart. In this study, we tested the effects of acetate on contractile function of isolated mouse ventricular myocytes.

**Results:**

Acute exposure of myocytes to 10 mM sodium acetate caused a marked, but transient, decrease in systolic sarcomere shortening (1.49 ± 0.20% vs. 5.58 ± 0.49% in control), accompanied by a significant increase in diastolic sarcomere length (1.81 ± 0.01 μm vs. 1.77 ± 0.01 μm in control), with a near linear dose response in the 1–10 mM range. Unlike palmitate, acetate caused no change in action potential duration; however, acetate markedly increased mitochondrial Ca^2+^ uptake. Moreover, pretreatment of cells with the mitochondrial Ca^2+^ uptake blocker, Ru-360 (10 μM), markedly suppressed the effect of acetate on contraction.

**Conclusions:**

Lehninger and others have previously demonstrated that the anions of weak aliphatic acids such as acetate stimulate Ca^2+^ uptake in isolated mitochondria. Here we show that this effect of acetate appears to extend to isolated cardiac myocytes where it transiently modulates cell contraction.

## Background

It is well established that the cardiac myocardium is capable of oxidizing a variety of carbon sources to supply the energy required for continuous contraction. Lipids, carbohydrates, ketone bodies, and amino acids can all support some degree of ATP synthesis in the heart. The loss of metabolic flexibility in the diseased heart may lead to abnormal contractile function. For example, we recently demonstrated that mice overexpressing fatty acid transport protein (FATP4) in the heart have impaired diastolic function [1]. Similarly, acute exposure to long chain fatty acids has been shown to cause a decrease in cardiomyocyte contractility through effects on increases in voltage gated K^+^ currents thus causing shortening of the action potential [2].

In a continued effort to understand the relationship of cardiac metabolism and cell function, we set out to test the effects of acetate on contraction. Several studies have examined the effect of acetate on cardiac contraction with equivocal results. In isolated cells, acetate tends to increase cell shortening after 10 minutes of exposure [3]. In isolated papillary muscle and in vivo, sodium acetate has been shown to reduce contractility [4,5], whereas other studies suggest that acetate causes an increase in contractility [6]. The effects of acetate on cardiovascular function in vivo are complicated by the concomitant vasodilatory effects that also must be considered [6]. These mixed results are consistent with the idea that acetate can affect cardiac contractility, but the cellular mechanisms remain poorly understood.

Although it is typically found in low concentrations (~0.2 mM) in non-ruminant mammals [7], acetate oxidation can account for ~10% of the total CO_2_ output in humans [8]. Acetate can be converted to acetyl CoA by acetyl CoA sythetase (AceCS2) in the mitochondrial matrix [9] and the resultant acetyl CoA can then enter the tricarboxylic acid (TCA) cycle. The heart is unique in that the expression of the mitochondrial AceCS2 is higher than in any other tissue, so the heart is ideally suited to use acetate as a fuel source [9]. Metabolic studies by Randle demonstrate that acetate is rapidly oxidized in the myocardium [10]. In some isolated heart studies, acetate combustion can account for ~90% of total respiration to the exclusion of glucose oxidation [11], although others suggest that at physiological workloads both acetate and glucose are effectively utilized [12]. Acetate can also affect isolated mitochondria independent of its oxidation, where Lehninger and others have demonstrated that exposure to acetate causes a rapid increase in mitochondrial matrix Ca^2+^ and osmotic swelling [13-15].

In this context, we investigated the effects of acetate on cardiac contractility in isolated cardiac myocytes. We tested the effects of acetate throughout a 10 minute exposure. The results demonstrate that acetate inhibits systolic function and increases cell relaxation within 2 minutes of exposure. The decrease in systolic function is transient, however, and contraction amplitude is restored within 10 minutes. These effects are independent of changes in action potential duration; however, the effects of acetate were inhibited by blockade of mitochondrial Ca^2+^ uptake with Ru-360, indicating that acetate causes effects on cardiac contraction by increasing Ca^2+^ uptake into the mitochondria.

## Methods

### Animal subjects

All animals used in this study were male, aged 2–4 months, C57Bl6/J. All procedures complied with the standards for the care and use of animal subjects as stated in the *Guide for the Care and Use of Laboratory Animals* (NIH publication No. 85–23, revised 1996). Protocols were approved by the USUHS Institutional Animal Care and Use Committee.

### Solutions (concentrations in mM)

Normal Tyrode Soution (NT): NaCl, 137; KCl, 5.4; NaH_2_PO_4_, 0.16; glucose, 10; MgCl_2,_ 0.5; CaCl_2_, 1.8; HEPES, 5.0; NaHCO_3_, 3.0; pH 7.35 - 7.4.

Wittenberg Isolation Medium (WIM): NaCl, 116; KCl, 5.3; NaH_2_PO_4_, 1.2; glucose, 11.6; MgCl_2_, 3.7; HEPES, 20; L-glutamine, 2.0; NaHCO_3_, 4.4; KH_2_PO_4_, 1.5; 1X essential vitamins; 1X amino acids; pH 7.3-7.4.

### Myocyte isolation

Isolation of ventricular myocytes was performed as described previously [1,2,16]. Briefly, mice were anesthetized by intra-peritoneal injection with 2,2,2 tribromoethanol (250 mg/kg). Following cervical dislocation, the heart was rapidly excised and the aorta cannulated. The heart was retrogradely perfused with Ca^2+^-free WIM solution for 5 minutes followed by perfusion with a digestion solution containing 100 μM CaCl_2_ and 1 mg/mL collagenase (Type 2, Worthington Biochemical). Left ventricular cells were gently dispersed by manual trituration using a pasteur pipette in WIM solution supplemented with bovine serum albumin (1 mg/mL) and 500 μM CaCl_2_. Cells were washed twice with WIM solution and twice with HEPES-buffered M199 solution and stored at room temperature. Cells were used for experiments within 12 hours of isolation in all cases.

### Myocyte contraction measurements

Unloaded sarcomere shortening was measured in freshly isolated ventricular myocytes, as described previously [1,2]. Briefly, isolated myocytes were transferred into a recording chamber mounted on an Olympus X51 inverted microscope and superfused with normal Tyrode solution saturated with room air. Additions to the Tyrode solution are described in the text. The mitochondrial calcium uptake inhibitor, Ru-360, was obtained from EMD Biosciences; all other chemicals were purchased from Sigma. Typically, cells were field stimulated to contract at 1 Hz. When thapsigargin was applied to cells, the stimulation frequency was reduced to 0.5 Hz. Video images were acquired using a Myocam camera and IonWizard software (IonOptix, Inc.). All experiments were performed at room temperature.

### Mitochondrial Ca^2+^ measurements

Freshly isolated ventricular myocytes were plated on laminin-coated (100 μg/mL) Mat-Tek dishes for fluorescence imaging. Cells were loaded with Rhod-2-AM (5 μM) for 30 minutes in normal Tyrode solution containing probenecid (500 μM) to inhibit dye export and 200 μM MnCl_2_ to quench cytoplasmic fluorescence as has been previously reported [17,18]. After loading, cells were washed twice with normal Tyrode solution supplemented with probenecid and 200 μM MnCl_2_ and transferred to the microscope stage. Cell images were obtained every 10 seconds for 10 minutes. Data were plotted as background-subtracted Rhod-2-AM fluorescence normalized to mean signal during the first 6 images recorded prior to addition of acetate.

### Action potential measurements

Action potentials were measured in freshly isolated ventricular myocytes using whole cell current clamp. Briefly, following acquisition of the whole cell mode, cell holding potential was adjusted to −70 mV using current injection. Action potentials were evoked by suprathreshold stimuli (2 nA, 3 msec) delivered at 1 Hz. Action potentials were recorded continuously during 5 minute exposure to acetate followed by 5 minutes washout. Average traces constructed from 25 consecutive action potentials during control, acetate exposure (2 minutes) and washout (5 minutes) were analyzed. Action potential duration (APD_90_) was determined at 90% repolarization and referenced to the peak of the action potential.

### Data analysis

All data were analyzed using ClampFit, IonWizard, ImageJ and Microsoft Excel software and (except where noted) results are presented as mean ± SEM (standard error of the mean). Statistical analysis was performed with built-in functions of Excel or with the Sigma XL software add-in. Statistical tests and p-values are denoted in the figure legend and text where appropriate.

## Results

### Acute exposure to acetate transiently impairs cardiac contraction and increases diastolic sarcomere length

**Figure 1:**
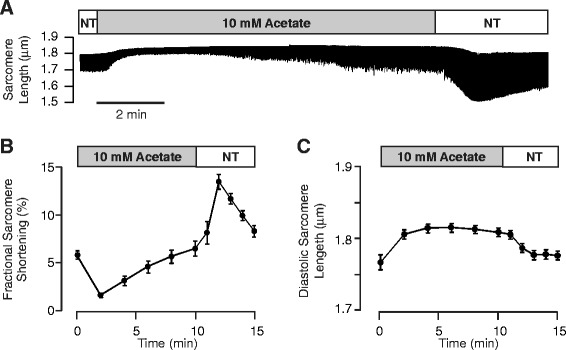
**Acetate causes a transient decrease of fractional sarcomere shortening and an increase in diastolic sarcomere length in isolated mouse cardiac myocytes. (A)** Representative recording of average sarcomere length assessed continuously throughout application and removal of normal Tyrode solution (NT) supplemented with 10 mM sodium acetate. Summary data from experiments as in **A** (n = 15), illustrating the effects of acetate on **(B)** fractional sarcomere shortening and **(C)** diastolic sarcomere length.

**Figure 2:**
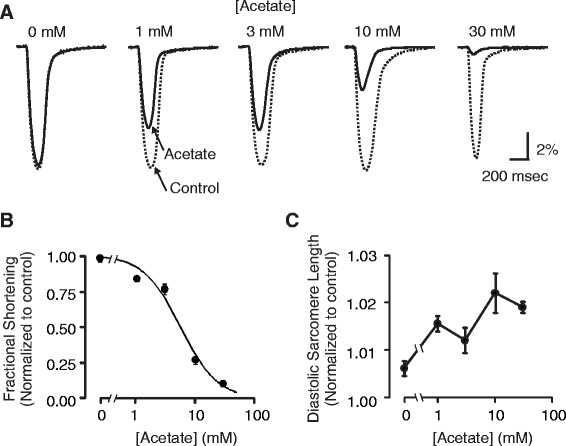
**Concentration-dependence of acetate effect on fractional shortening and diastolic sarcomere length. (A)** Single averaged contractions acquired in experiments as described in Figure 1 at different concentrations of sodium acetate. Contractions in normal Tyrode (Control, *dotted line*) and at 2 minutes following exposure to acetate solution (Acetate, *solid line*) are shown. 10 mM NaCl, instead of sodium acetate, was added to normal Tyrode to collect the zero acetate data. **(B)** Acetate concentration response curve for maximum contraction inhibition. Data were fit with a modified Hill equation (*solid line*): FS/FS_0_ = 1/(1 + ([Acetate]/IC_50_)^h^), where IC_50_ is the half-maximal inhibitory concentration of acetate (IC_50_ = 5.6 mM) and h is the Hill coefficient (h = 1.3). **(C)** There was no apparent acetate concentration dependence on diastolic sarcomere length.

To test whether the short chain fatty acid, acetate, exerts negative inotropic effects, we continuously monitored average sarcomere length in isolated mouse cardiomyocytes acutely exposed to Tyrode solution containing 10 mM sodium acetate. Three major consequences of acetate exposure were observed. Figure 1 shows that acetate caused a transient decrease in active sarcomere shortening. At two minutes following acetate application, fractional shortening was markedly decreased from 5.6 ± 0.5 to 1.5 ± 0.2 (n = 12, p < 0.001, paired t-test). In the continued presence of acetate, contraction amplitude gradually recovered and returned to baseline after approximately 10 minutes. We also noted a marked increase in fractional sarcomere shortening when acetate was removed from the bath solution. In addition, exposure to acetate significantly increased the diastolic sarcomere length. We next examined the concentration dependence of the decrease in contraction observed at two minutes and diastolic sarcomere length following the exposure to acetate. Figure 2 shows that the negative inotropic effect of acetate is concentration dependent. Data were fit with a modified Hill equation with an IC_50_ = 5.6 mM and Hill coefficient of 1.4. There was no apparent concentration dependence for the effect on diastolic sarcomere length.

### Acetate exposure does not affect the action potential duration

**Figure 3:**
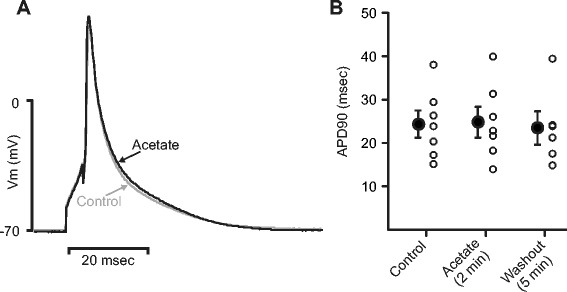
**Acetate does not affect the action potential duration (APD**_**90**_ **). (A)** Representative action potentials recorded before (Control, *gray line*) and 2 minutes following acetate exposure (Acetate, *black line*). Action potentials were recorded in whole cell current clamp mode and were evoked suprathreshold current injections. Records are averaged traces from 25 consecutive action potentials. **(B)** Summary APD_90_ data from all (n = 7) experiments as in **A**. Acetate exposure did not affect the APD_90_ (24.5 ± 3.2 msec in acetate vs. 25.0 ± 3.5 msec in control).

We previously demonstrated that acute exposure to long chain fatty acids shortens the action potential duration principally by increasing outward voltage-dependent K^+^ currents encoded by K_v_2.1 and K_v_1.5, with no effect on IK_1_ [2]. Considering that short-chain fatty acids might have similar effects on cell excitability, we examined the effect of acetate on the action potential duration (APD_90_) (Figure 3). In contrast to results with palmitate, APD_90_ (24.5 ± 3.2 msec in control) was unaffected by acetate (25.0 ± 3.5 msec, p >0.05, paired t-test), suggesting that a different molecular mechanism underlies the inotropic effects of acetate.

### Acetate exposure stimulates mitochondrial Ca^2+^ uptake

**Figure 4:**
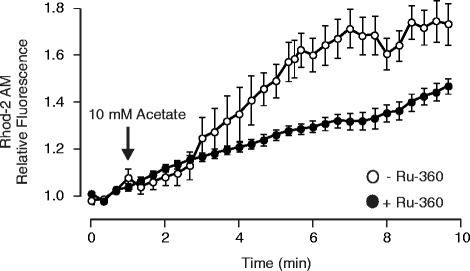
**Acetate stimulates mitochondrial Ca** ^**2+**^ **uptake.** Summary of rhod-2-AM fluorescence recorded during exposure to 10 mM sodium acetate in the presence (n = 8) or absence (n = 10) of Ru-360 (10 μM). Background-subtracted fluorescence was normalized to mean fluorescence during the period prior to addition of 10 mM acetate (n =10) or control (no acetate, n = 9). Acetate solution was added at the arrow.

It has been shown previously that acetate increases Ca^2+^ uptake in isolated liver and heart mitochondria [13-15]. In this light, we hypothesized that the acute application of acetate might lead to an increase in mitochondrial Ca^2+^ uptake, leaving less Ca^2+^ available to activate the myofilaments. To test this hypothesis, we first monitored mitochondrial Ca^2+^ during exposure to acetate using the fluorescent Ca^2+^ indicator, Rhod-2 AM (5 μM) in the presence of 200 μM MnCl_2_ to quench cytosolic fluorescence [17,18]. Figure 4 shows normalized cell fluorescence during exposure to 10 mM acetate in the presence or absence of the mitochondrial Ca^2+^ uptake inhibitor, Ru-360 (n = 9 and 8 respectively). This concentration of Ru-360 was chosen as it has previously been shown to have no effect on transmembrane Ca^2+^ fluxes other than mitochondrial uptake [19,20]. The results indicate that acetate causes a specific increase in Rhod-2 fluorescence consistent with the conclusion that acetate increases mitochondrial Ca^2+^ uptake.

### Inhibition of mitochondrial Ca^2+^ uptake attenuates the effects of acetate on fractional shortening and diastolic sarcomere length

**Figure 5:**
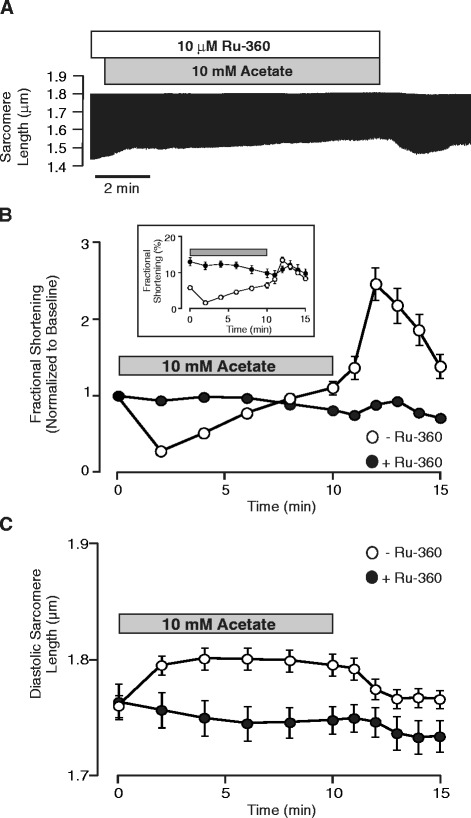
**Inhibition of mitochondrial Ca** ^**2+**^ **uptake with Ru-360 inhibits the effects of acetate on cell contraction. (A)** Representative recording of average sarcomere length assessed continuously throughout application and removal of normal Tyrode solution (NT) supplemented with 10 mM sodium acetate in cells pretreated with 10 μM Ru-360 for 30–60 minutes and maintained in Ru-360 throughout the acetate exposure. Summary data from experiments as in **A** (n = 11) illustrate that pretreatment with Ru-360 markedly suppresses the effect of acetate on both **(B)** fractional shortening and **(C)** diastolic sarcomere length.

If the acetate-dependent increase in mitochondrial Ca^2+^ is responsible for the reduction of myocyte fractional shortening, we reasoned that inhibiting mitochondrial Ca^2+^ uptake would reduce or abolish the effect of acetate on myocyte contraction. To test this hypothesis, we incubated cells for 30 minutes with 10 μM Ru-360, and then measured the effects of acetate on sarcomere shortening. Figure 5 shows that Ru-360 treatment significantly attenuated the effect of acetate on both diastolic sarcomere length and fractional sarcomere shortening. Interestingly, pretreatment with Ru-360 alone also had a marked effect on contractile function prior to acetate exposure. As shown in Figure 5B (inset), fractional sarcomere shortening immediately prior to acetate exposure (time zero) was 13.03 ± 1.17% in cells pretreated with Ru-360 compared with 5.83 ± 0.43% in control cells not exposed to Ru-360. Taken together, these data support the conclusion that acetate stimulates mitochondrial Ca^2+^ uptake, leading to reduced availability of Ca^2+^ for myofilament activation.

### Partial inhibition of SERCA inhibits recovery of systolic function during acetate application

**Figure 6:**
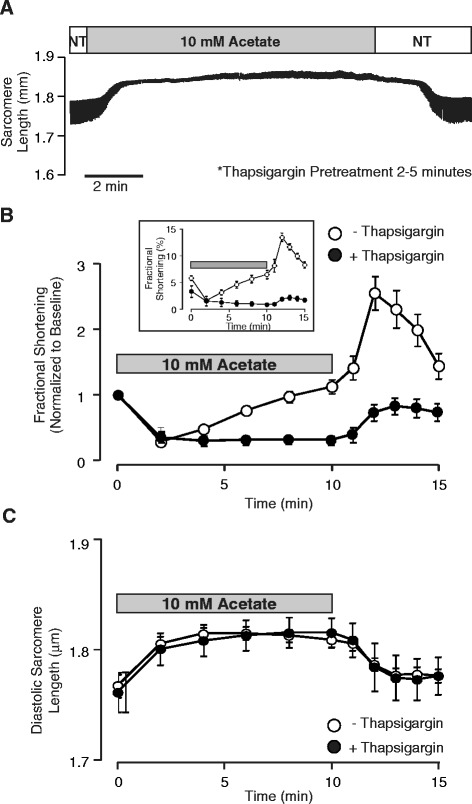
**Thapsigargin pretreatment specifically impairs the recovery of contraction during acetate exposure. (A)** Representative recording of average sarcomere length assessed continuously throughout application and removal of normal Tyrode solution (NT) supplemented with 10 mM sodium acetate in cells pretreated with thapsigargin (1 μM) for 2–5 minutes. As expected, cell relaxation was markedly slowed so contractions were evoked at 0.5 Hz. **(B & C)** Summary data from experiments as in **A** (n = 9). Thapsigargin pretreatment had no effect on the acetate induced decrease in fractional shortening or increase in diastolic sarcomere length, but markedly blunted the recovery of contraction typically observed from 2–10 minutes during acetate application.

The acetate-induced stimulation of mitochondrial Ca^2+^ uptake appears to be the seminal event leading to reduced inotropy. However, in the sustained presence of acetate, fractional shortening recovers to control levels by 10 minutes. This suggests that during the early phase of acetate exposure, cytosolic Ca^2+^ released from the SR bypasses the myofilaments and enters the mitochondria. We hypothesized that the recovery of contraction during the latter phase of acetate exposure reflects refilling of the SR to replace the Ca^2+^ lost to the mitochondria. Previous studies have utilized brief application of 1 μM thapsigargin to partially inhibit SERCA activity [21]. We therefore treated cells with 1 μM thapsigargin for 2–5 minutes prior to transfer to the bath for recording. No thapsigargin was added to the recording solutions. As expected, cell relaxation was markedly slowed by SERCA inhibition and therefore cells were stimulated at 0.5 Hz (instead of 1 Hz) for these experiments (Figure 6). Thapsigargin pretreatment markedly inhibited fractional shortening as expected (Figure 6B, inset), but exposure to acetate still resulted in a decrease in contraction amplitude and increase in diastolic sarcomere length similar to control, untreated cells. It should be noted, however, that the recovery of contraction typically observed during the latter phase of acetate application as well as the transient increase in contraction at washout were not observed in experiments where myocytes were briefly exposed to thapsigargin prior to acetate. This finding suggests that the recovery of contraction during acetate treatment reflects refilling of the sarcomplasmic reticulum with Ca^2+^.

## Discussion

### Acetate and cardiac function

There have been previous studies that have examined the effects of acetate on cardiovascular function and energetics in different contexts. Consistent with the results of the present study, acetate is reported to exhibit negative inotropic effects which appear to be transient [4,5]. It should also be noted that acetate infusion can also cause an acute reduction of the blood pressure that is most likely dependent on the increase in AMP associated with the conversion of acetate to acetyl CoA and local release of the vasodilator adenosine [6], complicating the interpretation of experiments in vivo. Nevertheless, the data in the present study indicate that acetate exerts effects on cardiac contraction by directly modulating myocyte function, at least partially independent of energy metabolism.

### A possible role for mitochondrial Ca^2+^ uptake in regulating contractile function

The present data provide evidence for a calcium-dependent mechanism linking acetate exposure with contractile function. Unlike the long chain fatty acid palmitate [2], the short chain fatty acid acetate caused no marked change in APD. Instead, we provide evidence that acetate caused a marked increased in mitochondrial Ca^2+^ accumulation. Moreover, the effect of acetate was markedly attenuated by pretreatment of myocytes with Ru-360, an inhibitor of mitochondrial Ca^2+^ uptake. Ru-360 is commonly used to inhibit mitochondrial Ca^2+^ uptake in a number of studies due to its ability to permeate cell membranes and it has been previously shown to have little if any effect on other transmembrane Ca^2+^ transport processes, including SR Ca^2+^ uptake and release, L-type Ca^2+^ channel and sodium calcium exchange function [19]. Our data is consistent with the conclusion that acetate uptake is coupled with an increase in mitochondrial Ca^2+^ and that this decreases, at least transiently, the amount of Ca^2+^ available for contraction of unloaded cardiomyocytes.

Interestingly, the effects of acetate on cell contraction were transient, with complete recovery of fractional shortening within 10 minutes of continuous exposure. This result could imply that as Ca^2+^ is sequestered in the mitochondria in the presence of acetate, additional Ca^2+^ enters the cell and refills the SR Ca^2+^ stores. We found that the acute decrease in contraction amplitude and increase in diastolic sarcomere length were unaffected by pretreatment with thapsigargin to attenuate SERCA activity. Interestingly, however, the *recovery* of depressed myocyte contractility during sustained acetate exposure and the abrupt increase in contraction amplitude due to acetate removal were no longer observed when cells were pretreated with thapsigargin. One possible explanation of this phenomenon is that inhibition of SR Ca^2+^ store replenishment prevents the restoration of fractional shortening.

### Acetate effects on mitochondria

Mitochondrial uptake of acetate has been shown to be coupled with increases in Ca^2+^ uptake and swelling [13]. It was proposed that direct transport of phosphate or anions of weak acids like acetate can permeate the mitochondrial membrane generatings a driving force for Ca^2+^ uptake into the mitochondrial matrix. Similar observations demonstrate that acetate or phosphate increase the rate of mitochondrial Ca^2+^ uptake in a concentration dependent manner [14] and that acetate increases mitochondrial Ca^2+^ uptake in heart mitochondria [15]. The data in the present study do not delineate the molecular transporters mediating Ca^2+^ entry into the mitochondria. In addition to the mitochondrial Ca^2+^ uniporter [22], the mitochondrial ryanodine receptor (mRyr) [23], or Ca^2+^/H+ exchanger (Letm1) [24] could play a role in the acetate-induced increase in mitochondrial Ca^2+^. Nevertheless, here we present pharmacological evidence showing that acetate is apparently coupled with an increase in mitochondrial Ca^2+^ in isolated cardiac myocytes and it is plausible to assume that this decreases the amount of Ca^2+^ available for contraction.

In isolated mitochondria, acetate also caused osmotic swelling [13]. Given the tight interfibrillar packing of mitochondria in the heart [25,26], acetate associated mitochondrial swelling might also be linked to the observed increase in diastolic sarcomere length. Interestingly, the effect of acetate on diastolic sarcomere length, unlike the effect on contraction, is sustained throughout acetate exposure and shows little concentration dependence. This observation is consistent with the conclusion that acetate causes the mitochondria to swell increasing the sarcomere spacing. While active cell shortening recovers as the SR refills with Ca^2+^, the mitochondria remain swollen and the increased diastolic sarcomere length is maintained. Given the structural constraints, it seems possible that swelling is physically limited possibly explaining the absence of a clear concentration dependence.

The observed delay from acetate application to initial effects on contraction is similar to the time course for conversion of acetate to acetyl CoA observed by Randle [10]. Acetate has been shown to decrease the phosphorylation potential [27] resulting in decreased free energy from ATP hydrolysis; since the demand likely remains constant, the cell must increase the rate of respiration to cover the difference. This might suggest that acetate oxidation increases mitochondrial respiration with concomitant increases in mitochondrial Ca^2+^. However, this is very unlikely since substrate availability governs only the pathways used to generate ATP, while workload or demand is the principal determinant of respiration [12,28]. In unloaded myocytes paced at a constant frequency, it is expected that the workload is constant; therefore, it is unlikely that acetate-induced changes in respiration underlie the increase in mitochondrial Ca^2+^ uptake. Rather, we consider the possibility that mitochondrial uptake of the acetate anion is electrically balanced by the uptake of Ca^2+^ in line with the conclusion of others based on experiments with isolated mitochondria [13-15].

Short chain fatty acids like acetate and butyrate may also cause changes in intracellular pH with effects on contraction and SR Ca^2+^ content [29,30]. The current data do not preclude a role for cytoplasm acidification in the effect of acetate on myocyte contraction. However, the observations that acetate increases mitochondrial Ca^2+^ and that pretreatment of cells with Ru-360 markedly attenuates the effects of acetate argues that an acute change in mitochondrial Ca^2+^ uptake, rather than cytoplasmic acidification, is the predominant mechanism underlying the effects of acetate on contraction. Moreover, it is tempting to predict that other anions of weak organic acids (e.g. lactate, butyrate, or pyruvate) may cause similar changes.

### Mitochondrial Ca^2+^ uptake and cardiovascular disease

In the heart, mitochondrial Ca^2+^ uptake has been proposed as an important player in regulating cardiac energetics, reactive oxygen species generation and supply–demand matching [31,32]; however, mitochondrial Ca^2+^ overload is also associated with the activation of cell death pathways [33]. Thus a balance in mitochondrial Ca^2+^ loading is required in order to achieve proper regulation of metabolism, but avoid overloading and cell death. In ischemia-reperfusion experiments, excessive Ca^2+^ uptake is associated with a poor outcome, and treatment with Ru-360 to inhibit mitochondrial Ca^2+^ uptake is beneficial [34]. Acetate in this setting would be predicted to have no effect or be detrimental, and this has been shown to be the case [3,35-37]. Conversely, the failing heart has been shown to have reduced mitochondrial Ca^2+^ resulting from increases in cytosolic Na^+^ concentrations and increased mitochondrial sodium-calcium exchange activity and blocking mitochondrial Ca^2+^ export (i.e. enhancing mitochondrial Ca^2+^) is beneficial [38]. The data in the present study suggest that elevating circulating acetate might be an alternative strategy to accomplish this goal, although it should be noted that the study presented here was focused on the transient and not steady state consequences of acetate.

## Conclusions

In summary, we have shown that acetate causes an acute but transient reduction in contractile function in isolated cardiac myocytes. Mechanistically, the transient negative inotropic effect appears to result from an acetate-dependent increase in mitochondrial Ca^2+^ uptake. This finding is consistent with the results of Lehninger and others using isolated liver and heart mitochondria, where it has been shown that acetate causes an increase in mitochondrial Ca^2+^ uptake and osmotic swelling [13-15]. Here we show that this effect appears to extend into intact, hydraulically unloaded cardiomyocytes, possibly suggesting a novel way to modulate mitochondrial Ca^2+^ homeostasis in the intact heart in vivo.
